# 肺癌患者的躯体化症状及与焦虑抑郁的相关性研究

**DOI:** 10.3779/j.issn.1009-3419.2017.07.06

**Published:** 2017-07-20

**Authors:** 心尧 张, 晓晔 张

**Affiliations:** 110004 沈阳，中国医科大学附属盛京医院第四肿瘤科 Department of the Fourth Medical Oncology, Sheng Jing Hospital, China Medical University, Shenyang 110004, China

**Keywords:** 肺肿瘤, 躯体化症状, 焦虑抑郁, Lung neoplasms, Somatization symptoms, Anxiety and depression

## Abstract

**背景与目的:**

肺癌是一种严重威胁人类健康的恶性肿瘤，其发病率及死亡率近年始终排在全国首位。肺癌患者常伴发焦虑、抑郁等情绪问题，而焦虑抑郁等情绪问题会进一步引发一系列躯体症状。目前，临床上对于肺癌患者的躯体化症状认识不足，相关的临床研究较少，本研究以肿瘤内科患者为研究对象，探究肺癌患者的躯体化症状及其与焦虑、抑郁的相关性分析。

**方法:**

对符合躯体化症状诊断标准的肺癌患者，用患者健康问卷躯体症状群量表（Patient Health Questionnaire-15, PHQ-15）中文版进行躯体症状统计，用汉密尔顿焦虑他评量表（Hamilton Anxiety Scale, HAMA）和汉密尔顿抑郁他评量表（Hamilton Depression Scale, HAMD）评定焦虑和抑郁状态。计算焦虑或抑郁检出率及不同程度躯体化症状人数，分析躯体化症状与焦虑、抑郁的相关性，统计不同躯体化症状发生频率的分布情况。

**结果:**

50例具有躯体化症状的肺癌患者中，存在焦虑抑郁情绪的患者有43例，仅焦虑、仅抑郁及焦虑合并抑郁的患病率分别为10%、10%、66%，其中躯体化症状程度越高，焦虑合并抑郁的检出率越高。躯体化症状与焦虑、抑郁的相关分析显示，PHQ-15总分、PHQ-15阳性症状数目与HAMA得分（*r*=0.752, *P* < 0.001; *r*=0.710, *P* < 0.001）、HAMD得分（*r*=0.648, *P* < 0.001; *r*=0.618, *P* < 0.001）呈显著正相关。具有躯体化症状的肺癌患者临床症状出现频率由高到低依次是疲劳（96%）、虚弱感（88%）、睡眠障碍（84%）、头晕（82%）、肢体或关节疼痛（80%）等；不同性别肺癌患者之间的躯体化症状比较差异无统计学意义（*P* > 0.05）。

**结论:**

具有躯体化症状的肺癌患者焦虑、抑郁常见，躯体化症状与焦虑、抑郁密切相关，此类患者最常见的临床症状为非特异性全身不适症状。

## Abstract (en)

**Background and objective:**

Lung cancer is a serious threat to human health and its morbidity and mortality in recent years has always been ranked first in the country. Lung cancer patients are often associated with anxiety, depression and other emotional problems, and anxiety, depression and other emotional problems will further lead to a series of somatic symptoms. At present, we lack the clinical understanding of somatization symptoms in patients with lung cancer and related clinical studies are not too much. This research is to study the features of clinical manifestation of lung cancer patients with somatization symptoms and correlative analysis between anxiety, depression and somatization symptoms in medical oncology department of general hospital.

**Methods:**

Lung cancer patients who met somatization symptoms diagnostic standard were assessed with the self-constructed somatic symptoms investigation questionnaire, the Chinese version of the Patient Health Questionnaire-15 (PHQ-15) and anxiety and depression were recorded by the Hamilton Anxiety Scale (HAMA) and Hamilton Depression Scale (HAMD). The detection rate of the anxiety, depression and the number of patients with different severity somatization symptoms were calculated, the relationship between the PHQ-15 scores and anxiety and/or depression and the distribution of the different frequency somatization symptoms were analyzed.

**Results:**

There were 43 patients with anxiety and/or depression in the 50 lung cancer patients with somatization symptoms. Prevalence rates of anxiety, depression and anxiety combined with depression were 10%, 10% and 66% respectively. The more severity the somatization symptoms are, the higher detection rates of anxiety combined with depression are. Significant positive correlations were observed between PHQ-15 scores, PHQ-15 positive symptom numbers and HAMA scores (*r*=0.752, *P* < 0.001; *r*=0.710, *P* < 0.001), HAMD scores (*r*=0.648, *P* < 0.001; *r*=0.618, *P* < 0.001). The most common somatization symptoms were fatigue (96%), weakness (88%), sleep disturbance (84%), dizziness (82%), and pain in extremities (80%). There was no significant statistical difference in somatization symptoms between lung cancer patients with different gender (*P* > 0.05).

**Conclusion:**

Symptoms of anxiety or depression were very common in lung cancer patients with somatization symptoms and there was a close relationship between anxiety, depression, and somatization symptoms. The most common symptoms of these patients were nonspecific generalized symptoms.

躯体化症状是临床中常见的，以多种多样、变化不定、反复出现的躯体症状为主要特征，即使患者患有某种确定的躯体疾病，但其存在的疾病并不能解释出现症状的程度和性质^[1]^。换言之，也就是指一个人有情绪问题或心理负担，但却通过各种躯体症状表现出来。肿瘤患者属于焦虑抑郁等情绪问题高发人群^[2, 3]^，各类肿瘤患者伴抑郁障碍的平均发病率为24%，其中，肺癌作为威胁全球人类生命健康的主要杀手之一，其发病率及死亡率近年始终排在全国首位。有研究^[4]^结果表明，肺癌的康复问题引起的心理痛苦以及心理需求未满足的情况多于其他肿瘤。情绪的变化经常引起一些临床无法解释的躯体化症状，这些症状复杂多样，可以累及多个系统，而反复主诉的躯体化症状又会进一步加重患者的焦虑抑郁情绪，从而导致患者反复就医和社会功能障碍。而临床医生往往对肿瘤患者合并躯体化症状的诊断缺乏认知，本研究以肺癌患者为研究对象，拟初步分析肿瘤患者躯体化症状的表现特点及其与焦虑、抑郁的相关性，希望给临床诊断提供一定的提示作用。

## 1 资料与方法

### 1.1 调查对象

研究对象来源于中国医科大学附属盛京医院第四肿瘤科住院肺癌患者50例，病例收集时间为2015年8月-2016年10月。入组标准为：①具备适当的沟通能力，无智力障碍等，有能力完成量表评定；②年龄≥18岁，有独立的认知和自主能力；③既往无精神病史；④既往无其他严重基础疾病；⑤既往无酒精药物依赖者；⑥有明确的病理诊断为肺癌（包括非小细胞肺癌和小细胞肺癌）；⑦存在各种各样的躯体化症状，其特征是自觉痛苦或导致重大功能损伤的躯体症状，同时具有关于这些症状过度和不恰当的思维、感觉和行为，未发现合理的躯体症状解释；⑧除外因肿瘤进展等器质性原因或因放化疗等治疗副反应引起的躯体化症状（由至少2名主治医师判断确诊，并由精神心理科专业医师进行定期访谈），所有入组者均在入组时签署知情同意书。本次调查共发出问卷53份，收回有效问卷50份，回收率94.3%。50例有效患者中，均为肺癌，男性29例，女性21例；年龄42岁-75岁，平均（58.58±7.99）岁。其中，7例合并糖尿病，10例合并高血压；1例陈旧性心梗病史10余年，1例冠心病史10余年，口服药物治疗未再发作；1例房颤发作病史合并十二指肠溃疡病史20余年，后未再复发。患者血糖、血压平稳，相关化验检查指标平稳，病情控制良好，请相关科室及心理科医师会诊评估其伴随疾病不会引起焦虑抑郁等情绪问题。

### 1.2 调查内容及方法

对符合躯体化症状诊断标准的50例肺癌患者进行下列评定：

#### 1.2.1 一般人口学资料

记录患者的一般资料，包括姓名、年龄、性别、既往病史和已有的诊断。

#### 1.2.2 焦虑抑郁情绪评价

在精神心理科医师协助下，对入组患者进行汉密尔顿焦虑量表（Hamilton Anxiety Scale, HAMA）和汉密尔顿抑郁量表（Hamilton Depression Scale, HAMD）问卷调查。HAMA和HAMD由Hamilton于1959年编制，分别用于评定焦虑和抑郁症状及其严重程度，是精神科临床和科研领域对焦虑和抑郁症状进行评定的应用最广泛的他评量表，具有良好的信效度，广泛应用于肿瘤临床^[5]^。

HAMA为他评量表，共14个条目，均为0级-4级评分，0代表无症状，4代表极重，总分范围0分-56分，其中总分≥14分评定为存在焦虑。

HAMD为他评量表，共24项，其中14项采用0级-4级评分，0代表无症状，4代表极重度；10项采用0级-2级评分，0代表无症状，2代表重度，总分范围0分-76分，其中总分≥8分评定为存在抑郁。

#### 1.2.3 躯体化症状严重程度评价

研究者向入组患者行PHQ-15问卷调查。患者健康问卷（Patient Health Questionnaire-15, PHQ-15）是由Spitzer编制的一个由15个条目组成的用于评定医院患者躯体症状严重程度的自评量表，评定被调查者1个月内存在的躯体症状。Zhang等^[6]^的研究显示，该量表具有良好的信度，Cronbach's α系数为0.83。钱洁等^[7]^在国内的研究也显示该量表具有良好的内在一致性信度。以上结果均提示，PHQ-15量表条目简单、操作性好，有效性强。根据症状的严重程度分为3级评分：0（没有困扰）、1（少许困扰）、2（很多困扰）。总分范围为0分-30分，其中，0分-4分为无躯体症状；5分-9分为轻度躯体症状；10分-14分为中度躯体症状；≥15分为重度躯体症状。同时统计阳性症状条目数，总分为0分-15分。

### 1.3 统计学分析

采用SPSS 19.0统计软件进行数据分析。使用一般统计学描述分析样本中不同程度躯体化症状的分布以及焦虑或抑郁检出率，使用χ^2^检验比较不同性别肺癌患者躯体化症状发生频率的差异，使用*Pearson*相关分析分析躯体化症状与焦虑、抑郁的相关性。*P*＜0.05为差异具有统计学意义。

## 2 结果

### 2.1 焦虑或抑郁检出率及不同程度躯体化症状人数统计

50例有效患者中，HAMA得分范围7分-34分，平均（17.52±5.51）分；HAMD得分范围3分-30分，平均（12.34±6.30）分。5例HAMA≥14分且HAMD < 8分，评定为仅存在焦虑，占10%；5例HAMD≥8分且HAMA < 14分，评定为仅存在抑郁，占10%；焦虑合并抑郁有33例，占66%；其余根据评分标准没有情绪问题，即既无焦虑也无抑郁，有7例，占14%。

50例有效患者中，按PHQ-15划界分评定为不同躯体化症状程度组，其中轻度组（5分-9分）14例，占28%；中度组（10分-14分）21例，占42%；重度组（15分-30分）15例，占30%。PHQ-15阳性症状条目数评定结果为（9.52±2.24）分。PHQ-15总分评定结果为（12.32±4.33）分。

### 2.2 不同程度躯体化症状组焦虑、抑郁及焦虑合并抑郁的频率分布

在不同躯体化症状严重程度分组中，各组焦虑抑郁共病的检出率均明显高于仅有焦虑或抑郁的检出率，其中躯体化症状程度越高，焦虑合并抑郁的检出率越高，见表 1。

**1:** 不同程度躯体化症状组焦虑、抑郁及焦虑合并抑郁的频率分布 The frequency distribution of anxiety, depression and anxiety combined with depression in patients with different severity somatization symptoms

Group	Anxiety	Depression	Anxiety combined with depression
Mild	2 (14.3%)	2 (14.3%)	4 (28.6%)
Moderate	3 (14.3%)	2 (9.5%)	15 (71.4%)
Severe	0	1 (6.7%)	14 (93.3%)

### 2.3 躯体化症状与焦虑、抑郁的相关分析

躯体化症状与焦虑、抑郁的相关性分析结果显示，PHQ-15总分、PHQ-15阳性症状数目与HAMA得分均呈显著正相关（*r*=0.752, *P*＜0.001; *r*=0.710, *P*＜0.001）；PHQ-15总分、PHQ-15阳性症状数目与HAMD得分也均呈显著正相关（*r*=0.648, *P*＜0.001; *r*=0.618, *P*＜0.001）。

### 2.4 不同躯体化症状发生频率分布

#### 2.4.1 肺癌患者躯体化症状分布频率统计

在50例有效肺癌病例中，常见的躯体化症状为“疲劳或无精打采”48例（96%），“一阵阵虚弱感”44例（88%），“睡眠有问题或烦恼” 42例（84%），“头晕”41例（82%），“胳膊、腿或关节疼痛”40例（80%），可以看出肺癌患者中最常见的躯体化症状均为非特异性的全身不适症状，而最少出现的躯体化症状为“性生活中有疼痛或其他的问题”和“痛经或月经期间其他的问题”等生殖系统症状，见表 2。

**2:** 肺癌患者躯体化症状发生频率统计 The frequency of somatization symptoms in patients with lung cancer

Sequence	Common somatization symptoms	*n*	%
1	Feeling tired or having low energy	48	96
2	Fainting spells	44	88
3	Trouble sleeping	42	84
4	Dizziness	41	82
5	Pain in your arms, legs or joints (knee, hips, *etc*.)	40	80
6	Nausea, gas, or indigestion	38	76
7	Headaches	36	72
8	Chest pain	35	70
9	Back pain	34	68
9	Shortness of breath	34	68
9	Constipation, loose bowels, or diarrhea	34	68
10	Feeling your heart pound or race	25	50
11	Stomach pain	22	44
12	Pain or problems during sexual intercourse	4	8
13	Menstrual cramps or other problems with your periods (women only)	0	0

#### 2.4.2 比较不同性别肺癌患者躯体化症状发生频率

在50例肺癌患者中，男性29例，女性21例。根据PHQ-15问卷调查结果，比较不同性别肺癌患者之间不同躯体化症状的发生频率，可以看出，不同性别肺癌患者躯体化症状发生率的分布趋势基本一致，其中常见躯体化症状均为“疲劳或无精打采”，“一阵阵虚弱感”，“睡眠有问题或烦恼”，“头晕”，“胳膊、腿或关节疼痛”等全身不适症状。不同躯体化症状之间比较，差异均无统计学意义，见表 3。

**3:** 不同性别肺癌患者躯体化症状发生率比较 Comparison of incidence rate of somatization symptoms in lung cancer patients with different gender

Items	Somatization symptoms	*n*			%		*P*
		Male	Female		Male	Female	
1	Stomach pain	13	9		44.8	42.9	> 0.05
2	Back pain	21	13		72.4	61.9	> 0.05
3	Pain in extremities	24	16		82.8	76.2	> 0.05
4	Menstrual cramps	0	0		0.0	0.0	> 0.05
5	Headaches	20	16		69.0	76.2	> 0.05
6	Chest pain	23	12		79.3	57.1	> 0.05
7	Dizziness	25	16		86.2	76.2	> 0.05
8	Fainting spells	26	18		89.7	85.7	> 0.05
9	Palpitation	15	10		51.7	47.6	> 0.05
10	Shortness of breath	20	14		69.0	66.7	> 0.05
11	Sexual problems	2	2		6.9	9.5	> 0.05
12	Constipation or diarrhea	22	12		75.9	57.1	> 0.05
13	Nausea, gas, or indigestion	22	16		75.9	76.2	> 0.05
14	Fatigue	28	20		96.6	95.2	> 0.05
15	Trouble sleeping	25	17		86.2	81.0	> 0.05

## 3 讨论

研究发现，在临床上以躯体症状就诊的患者中，仅有不到1/3可以找到明确的躯体病因，有1/3的患者是与心理因素紧密相关的躯体疾病^[8]^。大量调查显示，癌症患者焦虑、抑郁的发病率为10%-30%，其中一项*meta*分析结果显示，肺癌患者的抑郁发病率为11%-44%^[9]^，一些年轻、晚期或个别癌种的患者，焦虑、抑郁的发病率甚至达到50%以上^[10]^。不同于其他的患者群，肿瘤患者不但需要承受着生命威胁的压力，而且也需要背负一定的经济与社会压力，从而更易导致情绪压抑，进而产生某些与疾病本身不符，同时与心理因素密切相关的躯体化症状。因此，针对肿瘤患者的躯体症状主诉，我们在关注症状病因诊治的同时，更要关注症状本身，并在心理、情绪方面给予高度重视。

本研究结果显示，中国医科大学附属盛京医院第四肿瘤科入组的50例肺癌患者中，具有躯体化症状的男女比例为1.38:1，这一结果与既往研究提示女性躯体化障碍多于男性的结果不同，如挪威的一项HUNT-Ⅱ研究结果显示，具有躯体化症状的男女比例为1:1.31^[11]^；董丽平等^[12]^通过对90例神经内科躯体化障碍患者的研究发现，躯体症状女性较男性多见，男女比例为1:1.5。这一差异可能与本研究纳入均为肺癌患者有关，而肺癌患者男性发病率高于女性；还可能与本研究纳入病例数较少有关，未来可以增加病例数进一步观察男女比例变化。

心理因素与躯体化症状交互影响。在我们研究的50例肺癌患者之中，有38例（76%）合并抑郁症状，38例（76%）合并焦虑症状，33例（66%）既有抑郁又有焦虑症状，合并抑郁或焦虑症状达到86%，提示躯体化症状与焦虑抑郁存在很高的共患率，对于其中14%患者未发现明显焦虑抑郁，考虑与患者躯体化症状程度较轻，未表现出明显的焦虑抑郁情绪有关，对于这一部分患者日后可以进一步进行随访。关于躯体化症状与其他精神障碍疾病的共患现象，不少学者做过调查，通过对广州6家大型综合医院906例在心内科门诊就诊的患者进行评定，发现躯体症状与焦虑、抑郁密切相关，躯体症状程度越重，躯体症状条目越多，焦虑、抑郁相对危险度越高^[13]^。本研究以肺癌患者为样本，同样得出类似结论。肺癌患者躯体化症状严重程度与焦虑抑郁呈显著正相关，其中重度躯体化的肺癌患者全部存在抑郁、焦虑。结果说明，具有躯体化症状的肺癌患者普遍合并焦虑、抑郁等情绪问题，而出现与患者疾病本身不相符的躯体化症状，更多的是心理因素的一种躯体表现。本研究也发现，有一部分躯体化症状患者并不愿意承认自己身体不适是由心理问题引发的，也不重视相关心理问题，更不愿意配合治疗，他们认为情绪问题对自己影响不大，往往怀疑躯体不适的原因是原有疾病的恶化，认为治疗躯体疾病要紧。因此，很多肺癌患者受困于焦虑抑郁情绪而不自知，长期压抑的情绪会使他们出现各种各样的躯体不适症状，而这些原因不明，常规治疗效果不佳的症状，又会进一步加重患者的焦虑抑郁情绪，周而复始，形成恶性循环，极大地降低患者的依从性及继续治疗的信心，无形中也造成医疗资源的浪费。

目前已知各种心身疾病与精神压力和情绪因素有密切关系^[14, 15]^，特别是诸如恶性肿瘤之类的慢性疾病，然而肿瘤患者的心理问题却并没有得到足够的重视，除了患者自身的认知因素外，临床医生的重视不足也是原因之一。医生通常只满足于关注躯体疾病，而忽视精神心理问题，甚至对心理问题诊断认识不足。本研究统计了入组的50例肺癌患者常见躯体化症状发生频率，发现躯体化症状出现频率高的依次是疲劳、虚弱、睡眠障碍、头晕、肢体或关节疼痛等非特异性全身不适症状；进一步研究结果表明，不同性别肺癌患者之间的躯体化症状比较无明显差异（*P*＞0.05）。国内一项关于神经内科躯体化症状患者的调查^[12]^显示，症状出现频率高的依次是头晕、头痛、睡眠障碍、疲劳、心悸、腰痛等。Gureje等^[16]^调查结果显示头痛、心慌出现的频率最高。本研究与上述结果略有差异，其原因可能是病例均源于肿瘤科，患者的全身不适症状较为突出。疲劳、虚弱等属于肿瘤内科较常见的躯体化症状，其原因众多，临床上当患者叙述这些症状，而又不能明确找到病因时，躯体化症状的诊断应当在考虑之内。有研究^[17]^认为，当患者有3种以上长期存在的躯体不适的主诉，同时多次躯体检查后仍存在难以解释的躯体症状时应特别注意识别，并将焦虑和抑郁列为优先考虑的诊断，从而减少漏诊及误诊。目前国内外关于肿瘤患者躯体化症状的临床研究较少，本研究得出的结果，希望可以提高肿瘤医生对具有躯体化症状的肺癌患者这一特殊群体的认识，在临床诊断时考虑心理情绪因素导致躯体症状的可能，尽早明确患者的病因，对于心理问题及时进行药物或非药物治疗，从而减轻患者的困扰，提高肿瘤患者的生存质量。

本研究入组样本量较少、地点局限，有待加大研究规模，进一步从性别、年龄、收入、教育背景、肺癌分期、治疗情况等方面分层分析，得出更全面的研究结果。日后研究也可以考虑纳入其他类型肿瘤患者，进一步比较不同癌种躯体化症状患者临床症状之间的差异。

## References

[1] Dimsdale J, Creed F, Escobar J (2013). Somatic symptom disorder: an important change in DSM. J Psychosom Res.

[2] Shankar A, Dracham C, Ghoshal S (2016). Prevalence of depression and anxiety disorder in cancer patients: An institutional experience. Indian J Cancer.

[3] Amirifard N, Payandeh M, Aeinfar M (2017). A survey on the relationship between emotional intelligence and level of depression and anxiety among women with breast cancer. Int J Hematol Oncol Stem Cell Res.

[4] Li J, Girgis A (2006). Supportive care needs: are patients with lung cancer a neglected population?. Psychooncology.

[5] Greer JA, Traeger L, Bemis H (2012). A pilot randomized controlled trial of brief cognitive-behavioral therapy for anxiety in patients with terminal cancer. Oncologist.

[6] Zhang L, Fritzsche K, Liu Y (2016). Validation of the Chinese version of the PHQ-15 in a tertiary hospital. BMC Psychiatry.

[7] Qian J, Ren ZQ, Yu DH (2014). The value of the Patient Health Questionnaire-15 (PHQ-15) for screening somatic symptoms in general hospital. Zhongguo Xin Li Wei Sheng Za Zhi.

[8] Kroenke K (1997). Symptoms and science: the frontiers of primary care research. J Gen Intern Med.

[9] Massie MJ (2004). Prevalence of depression in patients with cancer. J Natl Cancer Inst Monogr.

[10] Grassi L, Caruso R, Nanni MG (2013). Somatization and somatic symptom presentation in cancer: A neglected area. Int Rev Psychiatry.

[11] Haug TT, Mykletun A, Dahl AA (2004). The Association between anxiety, depression, and somatic symptoms in a large population: The HUNT-Ⅱ Study. Psychosom Med.

[12] Dong LP, Zhao HN, Chen YZ (2011). Features of clinical manifestation in patients with somatization disorder. Lin Chuang Jing Shen Yi Xue Za Zhi.

[13] Ye RF, Geng QS, Ou LM (2013). Correlative analysis between anxiety, depression and somatic symptoms in outpatients in cardiology clinic. Ling Nan Xin Xue Guan Za ZHi.

[14] Tully PJ (2010). Theories of depression and anxiety and cardiovascular outcomes in psychosomatic medicine and behavioral cardiology. Psychosom Med.

[15] Brostow DP, Petrik ML, Starosta AJ (2017). Depression in patients with peripheral arterial disease: A systematic review. Eur J Cardiovasc Nurs.

[16] Gureji O, Obikoya B (1992). Somatization in primary care: pattern and correlates in a clinic in Nigeria. Acta Psychiatr Scand.

[17] Mussell M, Kroenke K, Spitzer RL (2008). Gastrointestinal symptoms in primary care: prevalence and association with depression and anxiety. J Psychosom Res.

